# Host size overrides maternal effects on the development of a secondary hyperparasitoid wasp

**DOI:** 10.1093/jisesa/ieaf004

**Published:** 2025-01-23

**Authors:** Xianhui Shi, Rieta Gols, Jetske G de Boer, Jeffrey A Harvey

**Affiliations:** Department of Terrestrial Ecology, Netherlands Institute of Ecology, Wageningen, The Netherlands; Laboratory of Nematology, Wageningen University & Research, Wageningen, The Netherlands; Laboratory of Entomology, Wageningen University & Research, Wageningen, The Netherlands; Department of Terrestrial Ecology, Netherlands Institute of Ecology, Wageningen, The Netherlands; Aeres University of Applied Sciences, Wageningen, The Netherlands; Department of Terrestrial Ecology, Netherlands Institute of Ecology, Wageningen, The Netherlands; Department of Ecological Science, Section Animal Ecology, VU University Amsterdam, Amsterdam, The Netherlands

**Keywords:** *Cotesia glomerata*, *Gelis agilis*, host quality, provisioning, venom

## Abstract

Unraveling the numerous factors that drive phenotypic variation in trait expression among animals has long presented a significant challenge. Whereas traits like growth and adult size are often heritable and are passed on from one generation to the next, these can be significantly affected by the quality and quantity of resources provided by one or both parents to their offspring. In many vertebrates, such as birds and mammals, parents raise their young until adult, providing food, shelter, and protection. On the other hand, in insects, there is often little or no parental care, and the young are left to fend for themselves. Despite that, some insects can enhance the growth of their offspring. In parasitoid wasps, for example, mothers inject biochemical factors, including venoms, teratocytes, and virus-like particles into the host that increase host quality by regulating the nutritional milieu. However, it is not known whether maternal size is positively correlated with host regulation. Here, we evaluate maternal and host size-related effects on the development of an asexually reproducing (= female only) secondary idiobiont ectoparasitoid, *Gelis agilis* on pre-pupae in cocoons of its host, the primary parasitoid, *Cotesia glomerata*. Females *G. agilis* from 2 adult size classes, “small” (mean 0.7 mg) or “large” (mean 1.2 mg), were allowed to parasitize cocoons of differing size along a continuum from ~1.2 mg to ~4.0 mg, and the body size and development time of their offspring were measured. In both body size classes of *G. agilis* mothers, there was a strong correlation between host size and offspring size. However, there was no effect of adult *G. agilis* size on this parameter: for a given host size, the size of *G. agilis* offspring did not differ between small and large mothers. Our results reveal that host quality is mostly pre-determined, irrespective of maternal size.

## Introduction

Understanding the many factors underpinning phenotypic variation in the expression of traits in animals has long been a major challenge for evolutionary biologists. Offspring phenotype in most organisms is mediated by genetically based traits inherited from both parents, as well as through parental provisioning. Whereas in many organisms, morphological and developmental traits are heritable and are passed on from one generation to the next, these can be significantly affected by environmental factors as well as the quality and quantity of resources provided by one or both parents to their offspring. In vertebrates, such as mammals and birds, parents may provide food and shelter for their offspring until they attain maturity (Clutton-Brock 1991, Gross 2005, Cockburn 2006, Balshine 2012). Parental (usually maternal) provisioning is also sometimes observed in invertebrates, such as insects, and other arthropods. In some spiders, for example, mothers increase offspring survival and fitness through extended guarding and progressive provisioning of prey for up to several weeks during the gregarious phase (Gundermann et al. 1988, Ruch et al. 2014, Yip and Rayor 2014). Another, more extreme mode of provisioning in some insects and spiders is matriphagy, whereby the mother sacrifices herself as food to her newly emerged offspring (Kim et al. 2000, Toyama 2001, Suzuki et al. 2005).

In many arthropods, newly emerged young are left to fend for themselves and thus there is no visually apparent parental care (Gilbert and Manica 2010). However, even when they do not provide food or directly care for their offspring after eclosion, mothers can still invest in physiological provisioning by providing metabolic resources toward, i.e., larger eggs that store more proteins or lipids and thus provide more nutrients for the developing embryo(s) (Beukeboom 2018). Furthermore, the timing and location of maternal oviposition may profoundly influence offspring fitness. Optimization models predict that mothers should choose the most suitable environment or resources for offspring development, a process known as the “preference-performance” or “mother knows best” hypotheses (Gripenberg et al. 2010). In herbivorous insects, for instance, the natal plant on which the mother lays her egg(s) may differ in terms of the amount and quality of resources available for her progeny (Knolhoff and Heckel 2014). Consequently, our understanding of parental care and provisioning for offspring needs to incorporate a wide array of behavioral, physiological, and ecological criteria.

One aspect of maternal physiological provisioning in insects that has thus far received less attention is how adult female size may affect progeny growth and development. If larger eggs are assumed to enhance offspring fitness by containing higher levels of nutrients, it follows that egg size may be positively correlated with adult female size. This has indeed been shown for some insect taxa (Berrigan 1991) but not for others (Church et al. 2019). Discrepancies in the body size-egg size correlation may occur because, instead of investing in larger eggs, larger females may have evolved to invest in producing more eggs, thus balancing selection between *per capita* offspring fitness and offspring number (Maeta et al. 1998, Church et al. 2019). Another important factor is that egg size may depend crucially on maternal diet (Fox and Mousseau 1996), revealing indirect effects on this parameter.

Parasitoid wasps are model systems to study how maternal provisioning may affect offspring development and fitness. Parasitoids are insects whose larvae develop in or on the bodies of other arthropods, usually other insects, whereas the adults are free-living (Godfray 1994). Unlike almost all other insects, parasitoids depend on finite resources contained in individual hosts that are often not much larger than the adult parasitoid for their development and reproduction (Godfray 1994). Life history traits in parasitoids are thus strongly influenced by host size and hence quality (Godfray 1994), and they have evolved mechanisms to maximize the utilization efficiency and allocation of limited resources to various fitness-related functions (e.g., growth and reproduction) (Harvey 2005, Jervis et al. 2008).

Venoms injected by ectoparasitoids have an array of complex effects on host physiology and metabolism. Their primary function was long thought to be the induction of long-term host paralysis and developmental arrest, thus preventing displacement of the parasitoid larvae through host activity or molting (Asgari and Rivers 2011). However, research with the pupal ectoparasitoid, *Nasonia vitripennis*, reveals that venom plays an important provisioning role by increasing host lipid levels and helping to release nutrients such as amino acids stored in host tissues that are otherwise inaccessible to the parasitoid larvae (Rivers and Denlinger 1995, Danneels et al. 2010, Moreau and Asgari 2015, Mrinalini et al. 2015, Alvarado et al. 2020). Nakamatsu et al. (2007) found that venom, in combination with a parasitoid-derived polydnavirus (PDV), that was injected into their caterpillar host by the gregarious endoparasitoid *Cotesia kariyai*, significantly increased levels of trehalose and lipids in the host hemolymph. Thus, parasitoid venoms can profoundly affect host nutritional quality.

Thus far, numerous studies have examined how host-related factors, such as size or stage at parasitism, affect adult parasitoid size (Godfray 1994, Harvey 2005, Jervis et al. 2023). In the vast majority of these studies, however, the body size of the parasitoid mother was completely ignored, and therefore it was not possible to determine if larger mothers are able to manipulate host quality more effectively than smaller mothers. Larger female parasitoids may produce larger eggs or inject higher concentrations of venom than smaller females, and this may enhance their ability to supplement or manipulate the quality and quantity of host resources. In this study, using “large” and “small” females, we compared the development of the solitary ecto-hyperparasitoid *Gelis agilis* Fabricius (Hymenoptera: Ichneumonidae, Cryptinae) in differently sized cocoons of its host, the primary parasitoid, *Cotesia glomerata* L. (Hymenoptera: Braconidae, Microgastrinae). Females of species in the genus *Gelis* have been shown to have low fecundities and are only able to carry only a few very large “anhydropic” eggs at a given time in their life (Harvey 2008, Visser et al. 2016, Harvey et al. 2019). A previous study (Harvey 2008) found a non-significant relationship between adult female size and fecundity in *G. agilis*. This contrasts with most studies in parasitoids that produce large numbers of small, hydropic eggs and where adult female size and fecundity are often positively correlated (Jervis et al. 2001, 2008).

As far as we know, this is the first study to examine the role of maternal size in an idiobiont parasitoid on offspring performance. *Gelis agilis* has distinctively large venom glands and injects venom into the host before oviposition. Previous studies with this association had shown that adult body mass in *G. agilis* was strongly correlated with host cocoon mass at oviposition (Harvey 2008, Harvey et al. 2015). However, in these studies, the age and body size of the mothers was not determined. Here, both parameters were controlled. Large females weighed, on average, ~80% more than small females.

The main aim of this study is to determine if maternal size affects offspring size when developing across a range of host (cocoon) sizes at oviposition. Thus, can larger mothers enhance host quality through provisioning more effectively than smaller mothers? We hypothesize that larger mothers (i) lay larger eggs than small mothers, and (ii) more efficiently regulating host quality by injecting higher amounts of venom and thereby benefitting offspring development. The intimate ways in which hymenopteran hyperparasitoids manipulate their hosts through provisioning is discussed.

## Materials and Methods

### Insects

*Gelis agilis* is an asexually reproducing ecto-hyperparasitoid (Harvey 2008) that parasitizes the pre-pupae and pupae in cocoons of other primary parasitoids including *Cotesia glomerata*. Like most other species in the genus, *Gelis agilis* is wingless and mimics ants behaviorally, chemically, and morphologically (Malcicka et al. 2015, Harvey et al. 2018). Adult female wasps destructively host‐feed (Jervis and Kidd 1986) to obtain proteins crucial for egg production before they can parasitize hosts (Harvey et al. 2012). *Gelis agilis* is an idiobiont hyperparasitoid that paralyzes the host during the oviposition sequence, thus preventing its further development (Godfray 1994, Harvey 2005, Pennacchio and Strand 2006). These hosts are ostensibly “static” resources that “remains in place.” By contrast, koinobiont parasitoids attack hosts that continue feeding, growing, and molting throughout the course of parasitism (Godfray 1994, Harvey 2005, Pennacchio and Strand 2006). Hosts parasitized by koinobionts represent potentially “dynamic” resources that “do not remain in place.”

*Gelis agilis* was originally collected in fields in the vicinity of Wageningen from cocoons of *C. glomerata* and were maintained in a climate cabinet at 10 ± 2 °C under a 16: 8 h L: D regime with a relative humidity of 50% at the Netherlands Institute of Ecology (NIOO-KNAW). For rearing and experimental work, all parasitoids were maintained at 22 ± 2 °C under a 16: 8 h L: D regime with a relative humidity of 50%. Cocoons of the primary endoparasitoid *C. glomerata* provide suitable resources for *G. agilis* for host-feeding and progeny development. *C. glomerata* is a gregarious koinobiont endoparasitoid that attacks young larvae of the large cabbage white butterfly, *Pieris brassicae* L. (Lepidoptera: Pieridae), and other pierids. A single *C. glomerata* female lays up to 50 eggs per host (Harvey 2000). Parasitized caterpillars feed and grow until the final instar when the mature parasitoid larvae perforate holes in the cuticle, emerge, and spin cocoons. *Cotesia glomerata* were reared on first‐ or second‐instar larvae of *P. brassicae*, which were obtained from the general insect rearing of the Laboratory of Entomology at Wageningen University, the Netherlands. The larvae of *C. glomerata* take approximately 2 wk to develop inside their host depending on the temperature before they emerge from the host and spin cocoons.

Parasitized *P. brassicae* hosts were maintained in rearing cages (100 × 60 × 60 cm) and provided daily with fresh Brussels sprout plants (*Brassica oleracea*) as food until the parasitoid larvae egressed from their hosts and spun cocoons. Cocoon clusters < 24 h-old were collected and presented to adult *G. agilis* in cages (35 × 35 × 35 cm) for 3 d in which they were provided with honey and water. After that, the cocoons were collected and transferred to Petri dishes (ø = 12 cm) until eclosion of either *C. glomerata* or the hyperparasitoids. Newly emerged hyperparasitoids were collected and placed in cages as described above. Some wasps were used for the experiments or rearing. *Gelis agilis* females can produce eggs within 2 to 3 d following host‐feeding.

### Experimental Design

**Fig. 1.**
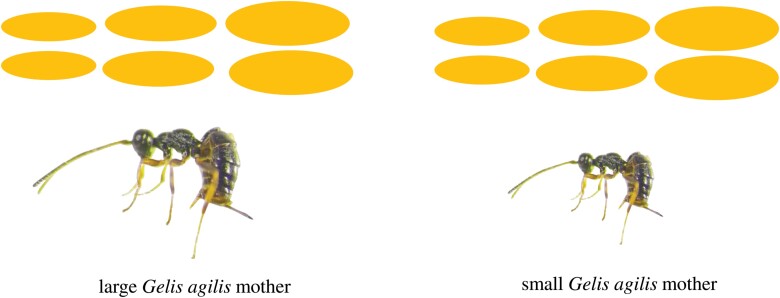
The experimental design showing different sizes of *Cotesia glomerata* host cocoons offered to large and small *Gelis agilis* mothers in a random size order in consecutive days.

Previous research has shown that *G. agilis* females vary in size between 0.5 mg and 1.8 mg when developing on differently sized cocoons of *C. glomerata* (Harvey 2008). Based on this, newly emerged females weighing 1.2 mg or more (representing “large” females) and 0.7 mg or less (representing “small” females) were used for the experiment. Females used for this experiment were collected as adults at emergence from *C. glomerata* cocoons. The aim was to ensure that the body size ratio between large and small females approached 2:1. Fifty small and 50 large *G. agilis* females were selected, weighed on a microbalance (Mettler-Toledo, accuracy 1 μg), and then placed individually into small Petri dishes (ø = 5.5 cm). Female *G. agilis* were initially narcoticized by exposing them to CO_2_ for ~10 min. Honey droplets and water-saturated cotton wool were then supplied in the Petri dishes as an energy and water source, respectively. Size variation of host cocoons was generated by collecting multiple broods of *C. glomerata* cocoons and separating them carefully using forceps and a cecum. Six *C. glomerata* cocoons were placed in each Petri dish to allow *G. agilis* to host-feed. When the females were ‘primed’ with mature eggs (this takes approximately 48 h), these cocoons were removed and each small or large *G. agilis* was offered individual cocoons of *C. glomerata* < 24 h old over 6 consecutive days (*G. agilis* females were, therefore, all between 3 and 8 d old) (Fig. 1). Freshly separated cocoons for the parasitism assay were weighed on the microbalance. Oviposition assays were conducted between 0900 and 1700 hours and parasitism was visually determined. Parasitized cocoons were then placed individually in small vials labeled with the mother’s ID and cocoon mass. After 10 d, host cocoons were checked several times daily for the presence of newly emerged wasps. Adult fresh biomass (described above) and development time in days were recorded. The fate of cocoons that did not produce *G. agilis* or *C. glomerata* were recorded as ‘dead cocoons. As a control, the fate of 40 unparasitized *C. glomerata* cocoons that differed in size (1.0 to 4.0 mg) was determined. To measure the egg length, 30 *G. agilis* females were selected along a size continuum (0.5 to 1.4 mg) and each was offered with 3 cocoons for host-feeding for 2 d. Then these *G. agilis* were dissected, and the length of the eggs was measured to the nearest μm using a Leica M205 C microscope. Egg length was determined for one egg per female.

### Statistical Analysis

To determine to what extent mother and host size determine offspring biomass 2 mother-size cohorts were established, one with females weighing less than 0.7 mg and one with females weighing more than 1.2 mg. A linear regression model (LM) was used to investigate whether the relationship between the host cocoon and offspring size differed for the 2 cohorts. In addition, we compared the oviposition duration of the 2 female size cohorts using Welch’s *t*-test as assumptions of equal variance were violated. Kaplan–Meier survival analysis was conducted to compare egg-to-adult development times of offspring from small and large mothers. Pearson’s chi-squared tests were used to compare the fate of cocoons when they were exposed to small or large females. Because there were no primary wasps emerging when large females were provided with host cocoons, this class was omitted from the contingency table analysis (Contingency table analysis requires that each class contains at least 5 items). A generalized linear model (GLM) was used to assess the effects of maternal size, cocoon size, and their interaction on cocoon fate (death, parasitism, or host emergence). ANOVA with Chi-squared tests was used to evaluate the main effects and interaction. All analyses were performed using R statistical software, version 4.3.3. (R Core Team 2024). Data and code generated in this study are available via the Dryad Digital Repository.

## Results

**Fig. 2.**
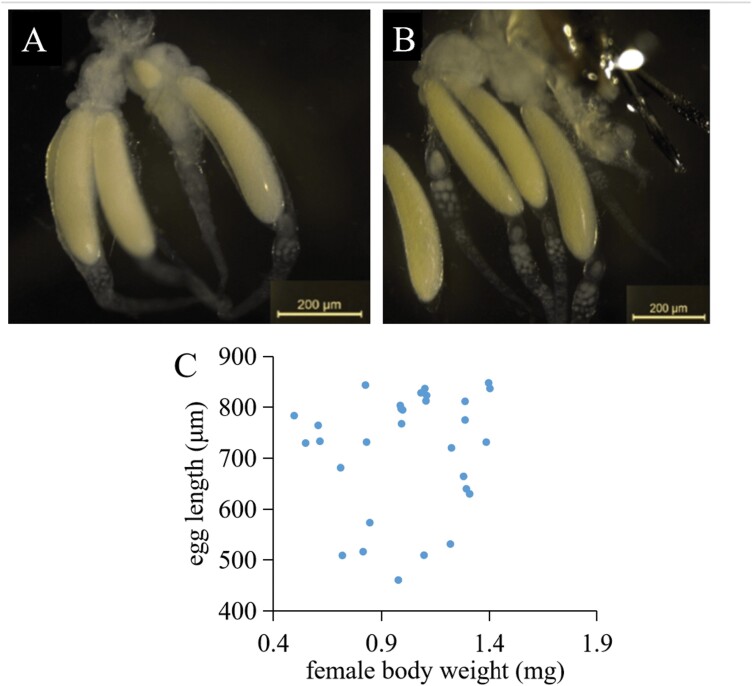
Dissection of *Gelis agilis* females showing 3 (A) and 4 (B) mature eggs from each *G. agilis* mother. (C) Scatterplot of egg length vs. *G. agilis* mother weight (linear regression: *F*_1, 28_ = 0.42, *P* = 0.52). Females were selected (from the rearing) along a size continuum from 0.5 to 1.4 mg (*n* = 30).

*Gelis agilis* produces large, yolky (= anhydropic) eggs that are produced in only very small numbers that are stored beneath the ovaries. Most females carry only one or 2 eggs at a given time, although in some wasps, there can be as many as 4 (Fig. 2A, B). There was no relationship between the size of the mothers and the length of their eggs (linear regression; *F*_1, 28_ = 0.42, *P* = 0.52; Fig. 2C), or between oviposition duration of large (30 ± 3 min, mean ± SE) and small (36 ± 7 min) mothers (*t*_62.1_ = 0.78, *P *= 0.43).

**Fig. 3.**
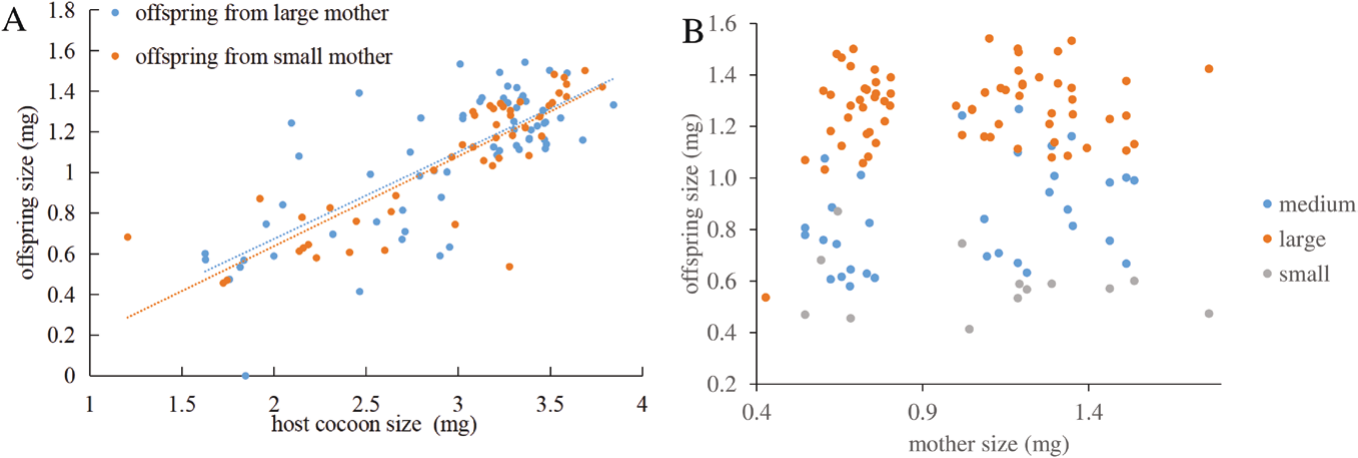
Linear relationship between *Gelis agilis* offspring size and cocoon size from large and small *G. agilis* mothers (A). There were no significant differences between the 2 lines (intercepts: *F*_1, 109_ = 0.10, *P* = 0.75; slopes, *F*_1, 109_ = 0.28, *P* = 0.59). For reference, the data are also presented with original mother sizes on the x-axis (B).

**Fig. 4.**
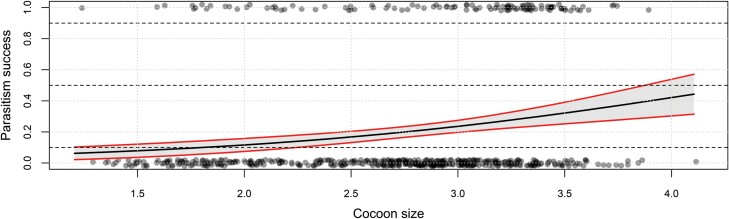
Proportion successful adult emergence of *Gelis agilis* when parasitizing host cocoons of different sizes (here depicted as biomass). The solid black line gives the predicted relationship based on the statistical model (see text) with a 95% confidence interval in gray and red.

**Fig. 5.**
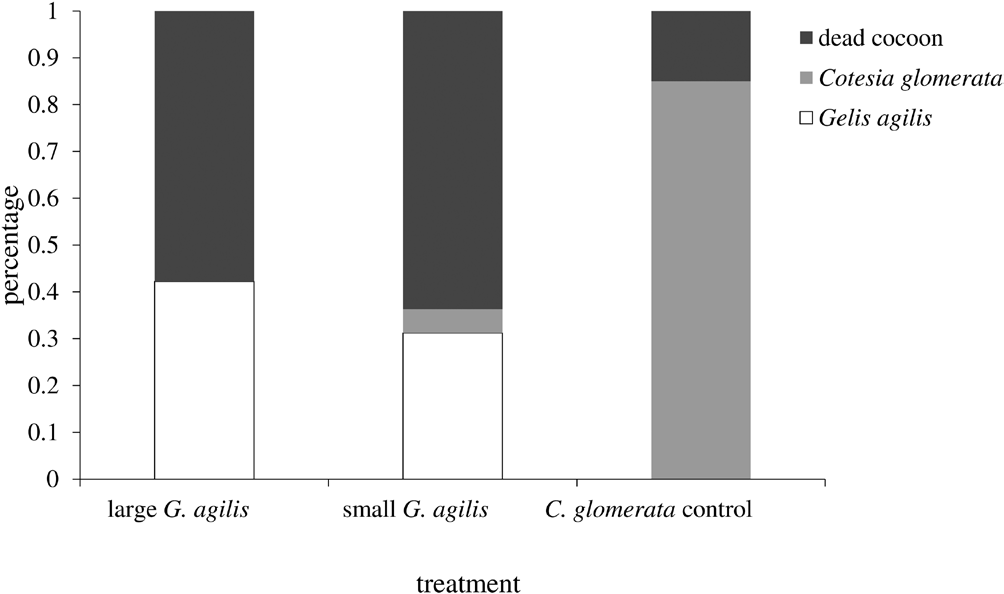
Fate of *Cotesia glomerata* cocoons when visually stung by large and small *Gelis agilis* mothers compared with controls (cocoons not presented to *G. agilis* females).

Body size of *G. agilis* offspring increased linearly with cocoon size at parasitism (*F*_1, 109_ = 317, *P* < 0.001, Fig. 3). Size of the mother did not affect the slope (*F*_1, 109_ = 0.28, *P* = 0.59) or the intercept of this relationship (*F*_1, 109_ = 0.10, *P* = 0.75). In other words, the lines for the 2 mother-size cohorts overlap (Fig. 3). Interestingly, offspring from small mothers took slightly, but significantly, longer to develop into adulthood (Log-rank test: *c²*_*1*_ = 10.3*, P *= 0.04, small 17.1 ± 0.2 d, large 16.5 ± 0.2 d). The fate of the parasitized cocoons, when exposed to small and large hyperparasitoid females, is shown in Fig. 4. To determine host quality, we also monitored cocoons that were not exposed to hyperparasitoids but were otherwise maintained under the same conditions. Eighty-five percent of these cocoons produced adult *C. glomerata*, which strongly contrasts with results for cocoons presented to *G. agilis* (Fig. 4). Moreover, mortality was significantly higher in cocoons that were exposed to large compared to small hyperparasitoid females (*c²*_*1*_ = 17, *P* < 0.001) and primary parasitoids only emerged from cocoons exposed to small mothers (Fig. 5).

## Discussion

The results of this study revealed that large and small mothers of *G. agilis* successfully parasitized a wide range of host (*C. glomerata*) cocoon sizes. Oviposition-to-adult development time was significantly longer in the offspring of small than large mothers. The fate of host cocoons presented to large or small mothers differed significantly among the 3 groups. Egg size did not differ with the size of the mother. Furthermore, the body size of the *G. agilis* mother had little or no effect on the body size of her offspring. Offspring size increased linearly across a range of host cocoon sizes at parasitism, and there was almost complete overlap in the offspring size of large and small mothers on host cocoons of similar sizes. This reveals that host size, a proxy of quality, is by far the prime determinant of offspring size in *G. agilis* and that maternal physiological provisioning ability through egg size or venom injected into the host does not apparently increase with female size. Although we did not measure genetic parameters in *G. agilis*, we also believe that our results would show that host size is by far the most important parameter affecting offspring size, irrespective of maternal genotype.

*Gelis agilis*, like other ectoparasitoids, possesses a comparatively large venom gland, and injects venom into its host before oviposition. This leads to rapid host paralysis, and this is important for ectoparasitoids because hosts that continue to grow or which remain active during parasitism can displace the parasitoid larvae feeding on them. Unlike koinobont endoparasitoids, which usually lay tiny, “hydropic” eggs into their hosts, idiobiont ectoparasitoids like *G. agilis* generally lay large, “anhydropic” eggs that are attached to the surface of the host body (Jervis and Kidd 1986, Ralec 1995). Hydropic eggs in parasitoids are unique in that they possess an “extra-embryonic membrane” that enables the embryo to uptake proteins directly from host hemolymph (Grbić and Strand 1998). During embryogenesis, hydropic eggs may therefore increase dramatically in size, and this strategy allows koinobionts to carry large complements of eggs that can be laid rapidly into their host (sometimes in less than a second!) and enables koinobiont endoparasitoid to exploit abundant or easily-accessible hosts (Price 1972). Anhydropic eggs, on the other hand, must contain all of the necessary resources for embryological development, and this means that ectoparasitoids generally produce far smaller numbers of them. Moreover, they often take considerable time to lay, as they must be compressed considerably while passing through the lumen of the female ovipositor. *G. agilis*, for example, takes up to an hour, and sometimes even longer, to lay a single egg onto the host. Extended handling time might be costly if it reduces parasitism efficiency (Wu et al. 2011), so this is an area that merits further investigation.

It has been known for many years that ectoparasitoid idiobionts like *G. agilis* have evolved various ways of physiologically regulating host quality in ways that benefit offspring fitness. As molecular tools have improved rapidly over the past few decades, our understanding of the physiological function(s) of venom has improved dramatically. It is now possible to determine exactly how venoms produced by some ectoparasitoid species affect their hosts. For instance, genomic sequencing of the jewel wasp, *Nasonia vitripennis*, a pupal parasitoid of filth flies (Muscidae) has enabled the precise function of its venom to be elucidated (Danneels et al. 2010, Moreau and Asgari 2015, Sim and Wheeler 2016). For instance, *N. vitripennis* venom stimulates differential gene expression in the hemocytes in the pupae of its host, *Musca domestica* (Qian et al. 2013). The altered genes are mostly related to a range of biological functions including nutrition, host immunity, stress responses, and regulation of transcription/translation.

Body size and development time are important proxies of fitness in parasitoids and most other insects (Godfray 1994, Harvey 2005, Jervis et al. 2008, 2023, Chown and Gaston 2010, Beukeboom 2018). These parameters have been shown to affect demographic processes such as reproduction and longevity, as well as resource-finding and dispersal ability, extrinsic competition, mating success, and defense (Charnov et al. 1981, Gross 1993, Chown and Gaston 2010, Ode et al. 2022). In parasitoids, numerous studies have focused on the importance of host-related factors such as age or stage of development at parasitism, size, and nutritional status, on parasitoid development (Godfray 1994, Harvey 2005, Jervis et al. 2023). Parasitoids are under strong selection for optimal allocation and utilization of ostensibly finite host resources to different and potentially competing fitness functions such as reproduction and survival (Jervis et al. 2008). Because of this, host–parasitoid interactions are physiologically intimate and represent a “nutritionally integrated system” (Brodeur and Boivin 2004, Pennacchio and Strand 2006, Harvey and Malcicka 2016). A previous study (Harvey, 2008) found no significant relationship between adult female size and reproductive success in *G. agilis*. We, therefore, hypothesized that a potential extra benefit of size would be that larger *G. agilis* mothers may be better able to provision their offspring early in development than smaller mothers, perhaps by injecting larger quantities of venom. As with *N. vitripennis* and other parasitoids, *G. agilis* venom is likely to have multiple functions that include increasing the access of stored nutrients, such as lipids and amino acids, to their larvae. However, as our study has shown, small mothers and their progeny are clearly able to regulate and exploit a given amount of host resources as effectively as large mothers and their progeny.

In summary, our study shows that host size is by far the most important factor affecting offspring size in the solitary idiobiont hyperparasitoid, *Gelis agilis* and that this parameter is not correlated with any maternal size-related provisioning or regulatory abilities. Despite that, more studies are needed to examine the role of parental size in parasitoids on offspring development from both a provisioning and genetic perspective. Both areas have thus far been little studied, and trends can only be elucidated when much more data is available. Several studies have reported that some traits in parasitoids are heritable (Kraaijeveld et al. 1998, Olson and Andow 2002, Martinez et al. 2012, Wajnberg et al. 2012). A recent study by (Rasekh et al. 2022) found some evidence for heritability for offspring size in a solitary koinobiont endoparasitoid based on maternal and paternal size. Future studies with parasitoids should aim to examine both the influence of heritability and provisioning (nature versus nurture) in the evolutioin of developmental strategies.
